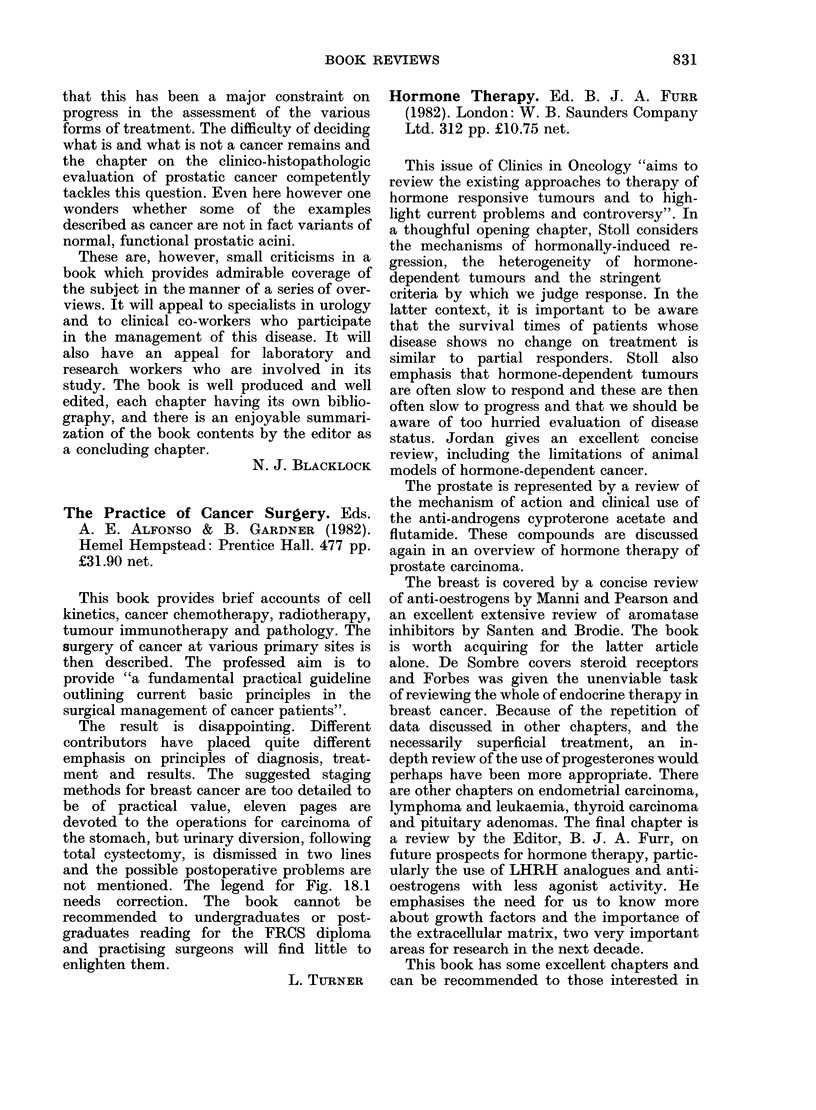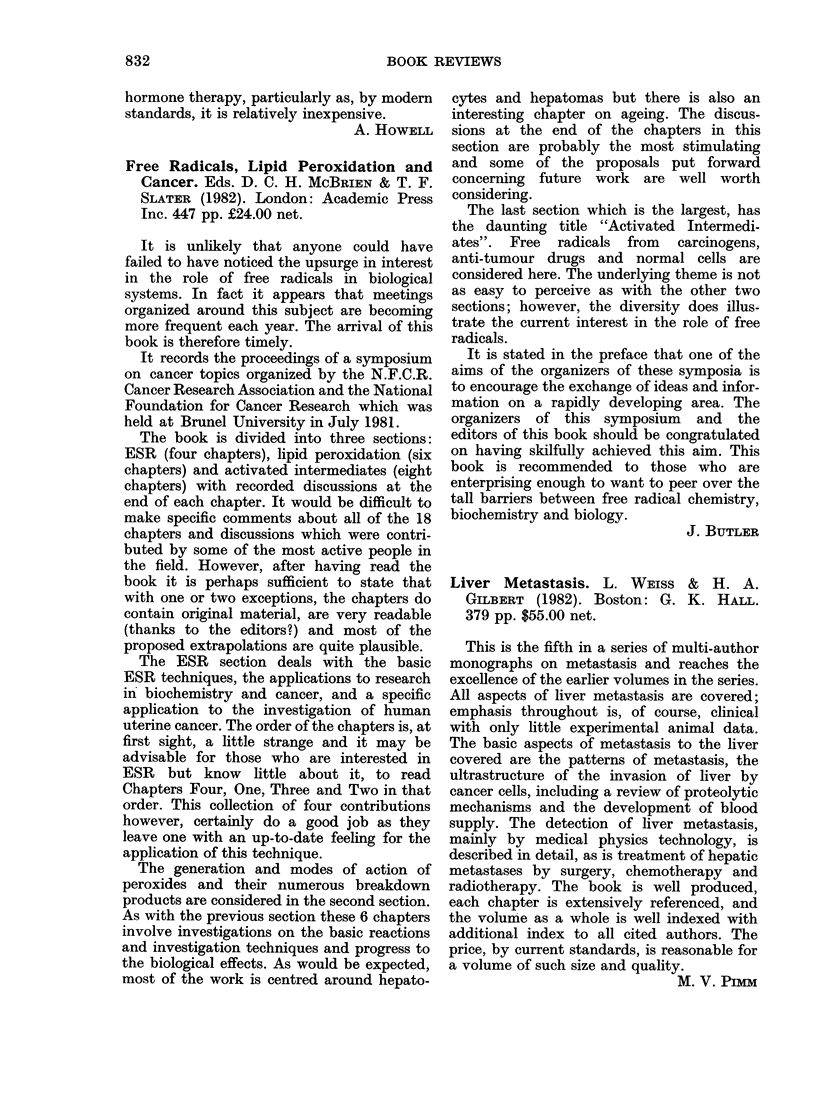# Hormone Therapy

**Published:** 1982-11

**Authors:** A. Howell


**Hormone Therapy.** Ed. B. J. A. FURR (1982). London: W. B. Saunders Company Ltd. 312 pp. £10.75 net.

This issue of Clinics in Oncology "aims to review the existing approaches to therapy of hormone responsive tumours and to highlight current problems and controversy". In a thoughful opening chapter, Stoll considers the mechanisms of hormonally-induced regression, the heterogeneity of hormone-dependent tumours and the stringent criteria by which we judge response. In the latter context, it is important to be aware that the survival times of patients whose disease shows no change on treatment is similar to partial responders. Stoll also emphasis that hormone-dependent tumours are often slow to respond and these are then often slow to progress and that we should be aware of too hurried evaluation of disease status. Jordan gives an excellent concise review, including the limitations of animal models of hormone-dependent cancer.

The prostate is represented by a review of the mechanism of action and clinical use of the anti-androgens cyproterone acetate and flutamide. These compounds are discussed again in an overview of hormone therapy of prostate carcinoma.

The breast is covered by a concise review of anti-oestrogens by Manni and Pearson and an excellent extensive review of aromatase inhibitors by Santen and Brodie. The book is worth acquiring for the latter article alone. De Sombre covers steroid receptors and Forbes was given the unenviable task of reviewing the whole of endocrine therapy in breast cancer. Because of the repetition of data discussed in other chapters, and the necessarily superficial treatment, an in-depth review of the use of progesterones would perhaps have been more appropriate. There are other chapters on endometrial carcinoma, lymphoma and leukaemia, thyroid carcinoma and pituitary adenomas. The final chapter is a review by the Editor, B. J. A. Furr, on future prospects for hormone therapy, particularly the use of LHRH analogues and anti-oestrogens with less agonist activity. He emphasises the need for us to know more about growth factors and the importance of the extracellular matrix, two very important areas for research in the next decade.

This book has some excellent chapters and can be recommended to those interested in hormone therapy, particularly as, by modern standards, it is relatively inexpensive.

A. HOWELL